# Effects of Brown Sugar Water Binder Added by Spraying Method as Solid Bridge on the Physical Characteristics of Biomass Pellets

**DOI:** 10.3390/polym12030674

**Published:** 2020-03-18

**Authors:** Kexin Zhang, Shuangyan Song, Zhongjia Chen, Jianbo Zhou

**Affiliations:** 1 Beijing Forestry University, Beijing 100083, China; Kexin_Zhang@bjfu.edu.cn (K.Z.); Song_shuangyan@bjfu.edu.cn (S.S.); 2 Forestry New Technology Research Institute, Chinese Academy of Forestry, Beijing 100091, China; 3 Beijing Forestry Machinery Research Institute of State Forestry and Grassland Administration, Beijing 100029, China

**Keywords:** biomass pellets, spraying method, densification, compressive strength, relaxation ratio, durability

## Abstract

The binder can improve the physical characteristics of biomass pellets by forming solid bridges and increasing the adhesion of biomass materials. Taking pine sawdust as raw material and brown sugar water with different concentration as a binder, this study adopted spraying and stirring methods, respectively, and mixed brown sugar water with biomass in diverse proportions. The characteristic of pellets such as durability, relaxation ratio and compressive strength were studied by orthogonal design. Through range analysis, BP (Back Propagation) neural network factor significance analysis and mapping the relationship between physical properties and factors according to the importance of each factor, the effect of densification variables on the physical characteristics of biomass pellets was studied, and the outcome of adding brown sugar water binder to raw material by spraying method in improving the densification quality of biomass was explored. Results showed the brown sugar water binder added to pine sawdust by spraying method could mix the binder and biomass raw material more evenly compared with the stirring method. The relaxation ratio of pellets obtained by spraying method was reduced by 13.47%. The optimal densification conditions of pine sawdust were when the compaction pressure was 100 MPa, the mass ratio of brown sugar to water was 2:1, the proportion of brown sugar water to biomass material was 3%, and the adding method was spraying.

## 1. Introduction

With the continuous evolution of industry and the improvement of people’s living standards, energy, as an essential material resource for human survival and social growth, has received more and more attention. Among them, fossil energy is still the primary source of energy supply in the world today. However, the non-renewable nature of fossil energy makes the sustainable development of global economy face the challenge of energy resource shortage. On the other hand, the air pollution, greenhouse gas emission and other ecological and environmental problems caused by fossil energy are becoming more and more severe [1]. Therefore, in order to improve energy conversion efficiency and reduce pollutant emissions [2], as a kind of green, pollution-free and abundant renewable energy, biomass energy has been paid more and more attention [3].

Biomass is a variety of organisms formed by photosynthesis, mainly from agricultural waste and agricultural and forestry processing waste, municipal solid waste and livestock manure [4]. Due to the properties of biomass from plants, such as low calorific value, small volume density and irregular physical shape, the collection, handling, transportation and storage of biomass are very difficult, which seriously limits the energy utilization of biomass. Biomass densification is one of the effective means to reduce above technical limitations [5,6]. Pellets provide an excellent energy density, thereby reducing transportation and storage costs and making them competitive with fossil fuels. Furthermore, pellets are more homogeneous in size and structure than the raw biomass, an advantage that facilitates automated feeding in continuous boiler systems [7].

In the 1930s, the United States successfully developed a spiral densification machine to study densification technology [8], and in the mid-1980s, the biomass fuel industry was gradually established [9]. Due to the abundance of biomass resources in China, the effective and rational utilization of biomass resources is one of the effective measures to alleviate the increasingly serious energy crisis and environmental pollution [10]. Since the 1980s, China has been committed to the research of biomass densification technology [11]. Since then, the densification mechanism has become a hot topic of biomass densification technology. Qi jing et al. observed rice husk biomass pellet by electron microscope and analyzed the densification mechanism of rice husk biomass pellet, including chemical and physical aspects [12]. Liu Shengyong et al. observed the micro-mechanism characteristic of biomass briquette, which used the electron microscope (JEM-2100F) to get the original images for analysis. The analysis of image indicates that density of briquette increased significantly, reducing the original porosity of biomass material. The structure of the densified briquette becomes superimposed vertically and horizontally, with less space between the layers [13]. However, biomass densification process is a very complex process of physical and chemical changes, most of the densification equipment does not only rely on large pressure for compression but also rely on binders to complete the densification process [14]. At present, the research of solid bridges has been paid much attention. Kaliyan et al. demonstrated through scanning electron microscopy (SEM) images and UV auto-fluorescence images that corn stover and switchgrass formed solid bridges mainly through their own natural binders [15]. Kong Lingjun et al. used waste wrapping paper fiber as binder to conduct biomass densification test. The results showed that the solid bridge formed by the binder improves the strength of the connection between particles, thus reducing negative effects of resiliencies on the densities and mechanical properties of pellets [16].

As binder can improve the particles adhesion, compressive strength and durability of biomass; reduce the densification pressure and the wear on production equipment and adjust temperature [17], it is an effective way to improve the quality of biomass pellets and reduce the energy consumption on compression to add the appropriate binder to the biomass raw material before densification [18]. However, some binders added to biomass raw materials may cause environmental pollution. Therefore, it is very important to make sure that a binder is environmentally friendly and cost-effective, along with being of high quality and energy efficient [19]. Brown sugar is an organic substance with high viscosity and easy combustion. At present, scholars have extensive research on brown sugar and other sugars as binders. Mallika Thabuot et al. showed that molasses as binder can increase the densification of biomass pellets [20]. Majid Soleimani et al. found that molasses showed better binder performance because it contained sucrose, monosaccharides, proteins and minerals [21]. Kaliyan et al. found that molasses as a highly viscous binder adhere to the surface of solid particles and acts as the solid bridge [22]. Mišljenovic et al. concluded that the soluble sugars contained in molasses can recrystallize after the drying processes and form solid bridges to capture more particles, which results in high mechanical strength of the pellets [23]. The results of Zhai Yunbo et al. show that the solid bridge formed by the agglomeration of molasses and lime can improve the compressive strength and impact resistance of biomass pellets [24]. Singh et al. reported that with the increase of the amount of molasses and sodium silicate binder added into the stubble, the strength of the briquette increased [25]. Nevena et al. studied the effect of the addition of syrup on the quality of wheat straw briquette, and the results showed that the addition of 1.5%~3% syrup can effectively improve the relaxation of wheat straw briquette density and compressive strength, and lower energy consumption. It is confirmed that syrup as a binder is effective in promoting densification at temperature lower than the glass transition temperature of wheat lignin [1].

The researchers found that binders help promote the densification of biomass and tested the effect of the ratio of binder addition on the physical properties of biomass pellets. However, in the literature, the binder is generally mixed with biomass raw material by stirring. The purpose of this study is to try to add binder by means of spraying in order to make the binder and biomass material mix more evenly than the traditional stirring method, so as to achieve a better densification effect.

In this paper, pine sawdust was used as raw material and brown sugar water as binder to study the effect of binder added by spraying method on the densification quality of biomass. Through orthogonal experiment, the physical characteristics of biomass pellets such as relaxation ratio, compressive strength and durability were analyzed to explore the effect of concentration, different proportion of binder addition and adding method of brown sugar water on the physical characteristics of biomass pellets and to find the optimal densification conditions of biomass based on brown sugar water as binder using spraying method. It provides a fresh way to further explore the preprocessing of different biomass and to optimize the optimal processing parameters.

## 2. Materials and Methods

### 2.1. Materials

Pine sawdust from Heilongjiang province was selected for this study. Considering that the moisture content of pine sawdust is affected by temperature and humidity, the sawdust is dried and adjusted to 12% moisture content and sealed for storage before the experiment. Brown sugar is in powder form made of pure sugarcane juice.

### 2.2. Methods

#### 2.2.1. Experimental Equipment

**Figure 1:**
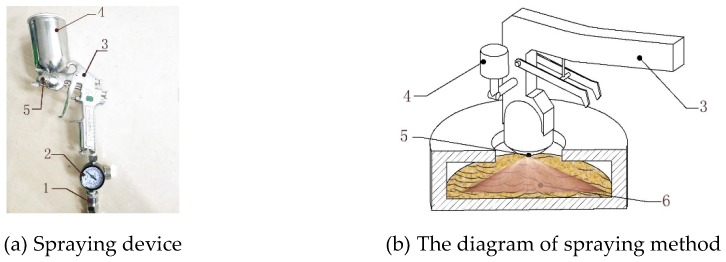
The spraying device: 1-Air inlet; 2-barometer; 3-Sprayer; 4-The pot of sprayer; 5-Nozzle; 6-Material container.

**Figure 2:**
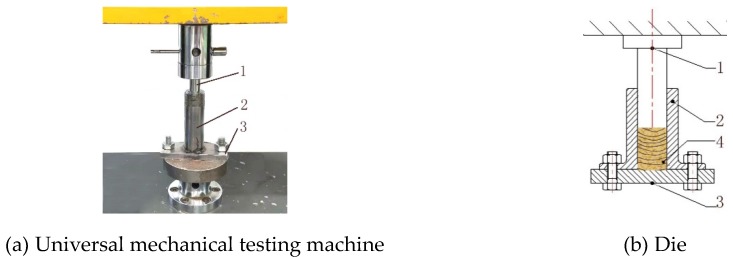
The schematic diagram of compaction apparatus: 1-Compressive bar; 2-Die sleeve; 3-Pedestal; 4-Biomass raw material.

Microcomputer-controlled electronic universal mechanical testing machine (Model: 4050, REGEER, Shenzhen, China), SC69-02 type moisture tester (Model: 5647, Shanghai, China), electronic balance (accuracy: 0.01 g), air compressor (Model: 750W-30L, Hangzhou, China), spraying device (Model: W-71-21G, Shanghai, China) (Figure 1), compaction die (inner diameter: 16 mm, length: 125 mm) (Figure 2), electronic caliper (precision: 0.01 mm), etc. The software includes IBM SPSS Statistics 24 (IBM, Armonk, NY, USA) and MATLAB 2018a (MathWorks, Natick, MA, USA).

#### 2.2.2. Pellet Preparation

Two means are employed to blend biomass raw material with binders before densification. The biomass raw materials and brown sugar water were put into the container 6 and the pot of sprayer 4 respectively, as shown in Figure 1. Air compressor was turned on to keep the outlet pressure of the sprayer at 4 MPa. Then the brown sugar water was spayed into biomass raw materials in the form of tiny liquid particles by pressing the handle of the sprayer. In this way, the binder particles produced by spraying method are expected to be fine and are blended with biomass evenly. The other way is to sprinkle brown sugar water on top of the raw material in the container 6 and then stir them for 5 min until the brown sugar water was comparatively distributed evenly among the biomass particles.

Mixture (7 g) is weighed with electronic balance and filled into the die. Then the universal mechanical testing machine is used to apply pressure to the compressive bar and compact the biomass material. After reaching the compaction pressure value (100 MPa) set in the test, the machine will stop and the pressure will be maintained for 30 s (retention time). The compressive bar was then withdrawn from the die at a rate of 5 mm/min. The densified biomass pellet was removed from the die, and the density was measured at once. Then pellet was placed in a sealed bag, and the physical characteristics were tested after 2 h (relaxation time).

#### 2.2.3. Test Index and Test Method

The relaxation ratio, durability (crushing strength) and compressive strength of biomass pellets was analyzed, mainly referring to the relevant literature and the series of standard methods of NY/T1881 test method for pellets of the Ministry of agriculture [26,27,28]. The diameter and length of the pellets with a relaxation time of 2 h were measured with an electronic caliper with a precision of 0.01 mm, and the relaxation density and relaxation ratio were calculated. In order to ensure the accuracy of the measurement, the average value and the standard error were calculated after diameter measurement from the three positions of biomass pellets. The mass of the biomass pellets was measured by electronic balance (0.01 g). Three samples were randomly selected from each group for each test. The formula (1) for calculating the relaxation density is as follows:

(1) $$\rho =\frac{4m}{\pi d^{2}L}$$

where:

ρ—relaxation density (g·cm^−3^);
m—mass of a biomass pellet (g);
d—end diameter of biomass pellet (cm);
L—length of biomass pellets (cm).

The formula (2) for calculating the relaxation ratio is as follows:

(2) $$\lambda =\frac{\rho_{m}}{\rho }$$

where:

λ—relaxation ratio (%);
ρ—relaxation density (g·cm^−3^);
$\rho_{m}$—maximum compaction density (the density of pellets when they were taken from the die) (g·cm^−3^).

Durability (crushing strength) reflects the ability of pellets to resist deformation after multiple falls and tumbling collisions [29]. According to the DB11/T541-2008 Beijing local standard testing method, the pellets are freely dropped from a height of 2 m to a flat cement floor for 5 times. The mass percentage of the pellets after the 5 drops, measured to be greater than 95%, indicates that the pellets have good quality. Three samples were randomly taken from each group for each test. The mass of the sample is measured before and after the test and the durability value calculated. In order to ensure the accuracy of the test, the average value and the standard error were taken for each group of test results. The durability calculation formula (3) is as follows:

(3) $$I=1-\frac{M_{b}-M_{f}}{M_{b}}\times 100\%$$

where:

I—durability (%);
$M_{b}$—mass before pellets drop test (kg);
$M_{f}$—mass after pellets drop test (kg).

The compressive strength is measured as follows: The axis of biomass pellet sealed for 2 h is placed vertically on the universal mechanical testing machine, and a little pressure is applied to ensure that the sample was fixed. When the test began, the compressive bar moved downward at a speed of 5 mm/min until the sample cracked completely [30]. The strength of the pellet was calculated from the maximum load, and the dimensions of the pellet, i.e., the pellet diameter and thickness [31]. The calculation formula of compressive strength (4) is as follows:

(4) $$\sigma_{t}=2\frac{F_{max}}{\pi Ld}$$

where:

$\sigma_{t}$—compressive strength (MPa);
$F_{max}$—maximum radial force (N);
d—end diameter of biomass pellet (cm);
L—length of biomass pellets (cm).

Three samples were randomly selected from each group for each test. In order to ensure the accuracy of the test, the average value and the standard error were taken for the test results of each group.

#### 2.2.4. Experimental Design

Using the mixed level uniform design table U_6_(3^2^ × 2^1^) to design a three-factor mixed level orthogonal test (Table 1), A, B and C respectively represent the mass ratio of brown sugar to water, the proportion of brown sugar water added and binder adding method. Y_1_, Y_2_ and Y_3_, respectively represent the compressive strength (MPa), relaxation ratio and durability (%). There were 6 sets of tests, each set of tests was repeated for 3 times, and the average value and the standard error were calculated. Since the main component of brown sugar is sucrose [32], and the solubility of sucrose at 25 °C is 202 g per 100 mL^−1^ [33], the mass ratio of brown sugar to water was selected in this experiment at three levels of 0:1, 1.5:1 and 2:1, respectively, among which level 1 was the control experiment, and level 3 was the mass ratio of the maximum solubility of brown sugar. Meanwhile, for pine sawdust, the optimal moisture content is between 11% and 13% [7]. Considering that high moisture content might make pellets too soft and fracture easily [34], and too many binders in industrial applications will increase the production cost, the brown sugar water addition ratio was selected as 1%, 2%, and 3%, respectively.

## 3. Results and Discussion

Raw data of the densified pellets are summarized in Appendix A Table A1 and the other experimental results are shown in Table 2. The average value K and range R of the physical properties of biomass pellets physical characteristics at each factor level were obtained (Table 3). According to the range analysis, the larger the range value is, the greater the influence of this factor on this index, and this factor is counted to be the main factor on the quality of biomass pellets. BP neural network in SPSS was used to analyze the significance of each factor to verify the results of range analysis. Secondly, the two factors that have the greatest impact on the index were selected according to significance, and the corresponding index and factor relationship diagram is drawn using MATLAB. Finally, the optimal equilibrium conditions of biomass pellets were obtained by using the comprehensive equilibrium method in the multi-factor analysis method.

### 3.1. Compressive Strength

**Figure 3:**
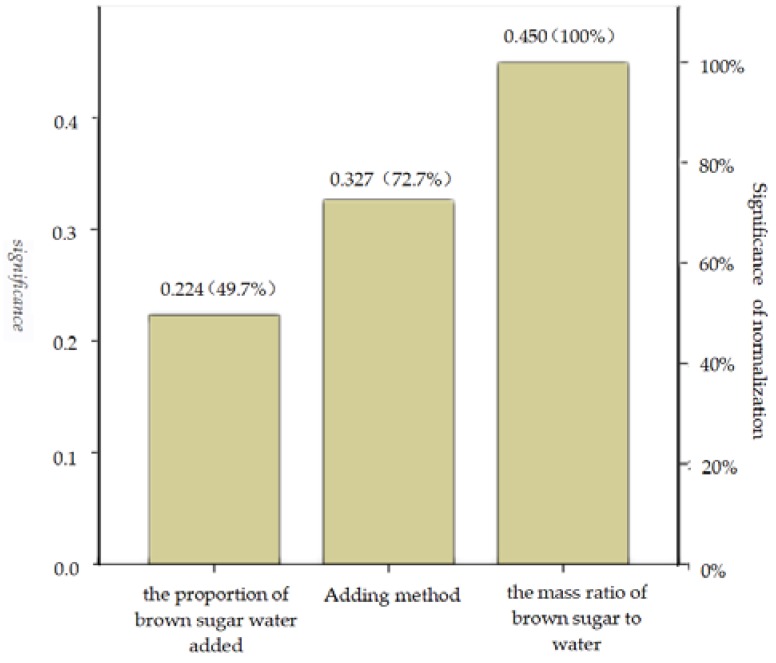
Chart of factor significance.

Table 3 shows the average value and the range of the compressive strength at different levels of each factor. According to the range analysis, the influence degree of each factor on the compressive strength was as follows: the mass ratio of brown sugar to water > binder adding method > the proportion of brown sugar water added. At the same time, the importance of each factor obtained using the BP neural network is consistent with the normalized significance (Figure 3) and the range analysis results.

**Figure 4:**
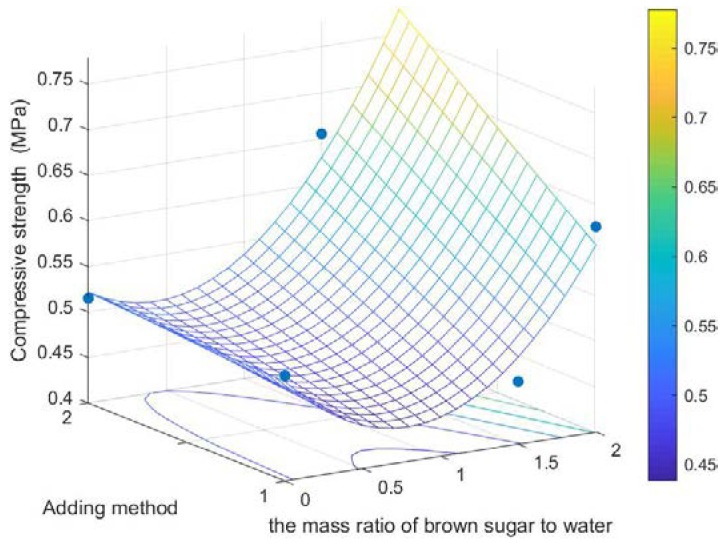
Relation diagram of compressive strength–the mass ratio of brown sugar to water and adding method.

It can be seen from Figure 3 that the normalized significance of the mass ratio of brown sugar to water and the binder adding method are 100% and 72.7%, respectively. The two major factors were selected, and the relationship between the compressive strength and the two major factors was plotted as shown in Figure 4.

**Figure 5:**
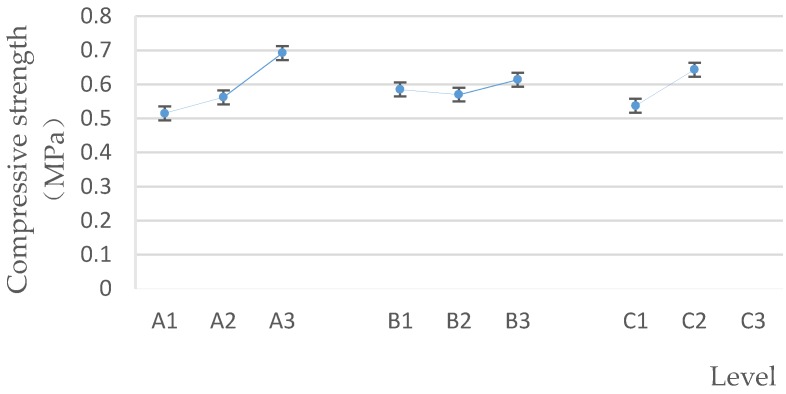
Factors and levels–compressive strength.

The higher the compressive strength is, the better the performance of biomass pellet will be. Thus, in the process of producing and transporting biomass pellets, the damage rate is reduced [30]. From the qualitative analysis shown in Figure 4, the higher the mass ratio of brown sugar to water and binder added by spraying, the greater the compressive strength within the specified factor level range. At the same time, according to the average value of each level of each factor (Figure 5), A_3_ was the optimal level of factor A, B_3_ was the optimal level of factor B, and C_2_ was the optimal level of factor C. Therefore, the optimal factors affecting the level of pellet compressive strength were A_3_B_3_C_2_; that is, the optimal pellet compressive strength is obtained as follows: the mass ratio of brown sugar to water is 2:1, the proportion of brown sugar water to raw material is 3%, and the binder adding method is spraying.

### 3.2. Relaxation Ratio

**Figure 6:**
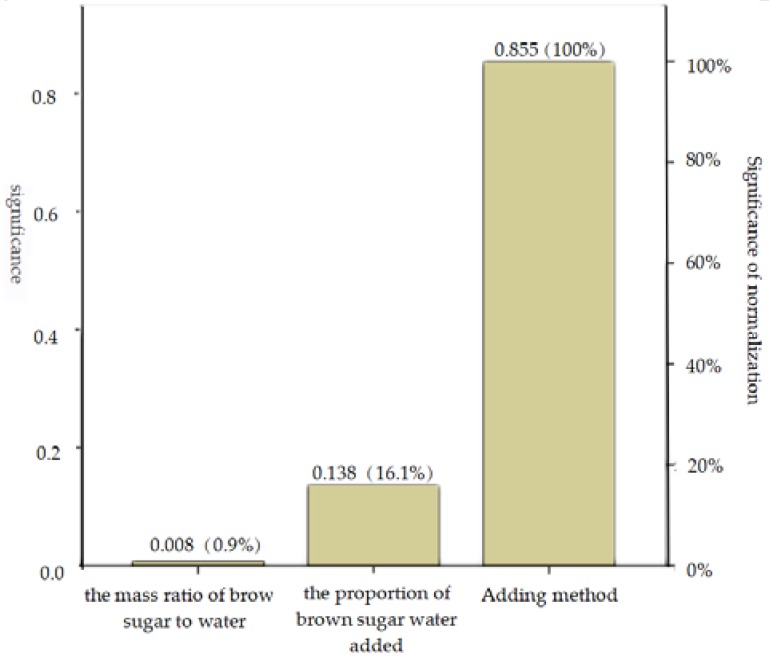
Chart of factor significance.

Table 3 shows the average value and range of relaxation ratio of each factor at different levels. According to the range analysis, the impact degree of each factor on the relaxation ratio was as follows: binder adding method > the proportion of brown sugar water to raw material > the mass ratio of brown sugar to water. That is, adding brown sugar water binder by spraying method has a significant effect on relaxation ratio of biomass pellets. The significance of each factor according to the BP neural network is consistent with the normalized significance (Figure 6) and the range analysis results.

**Figure 7:**
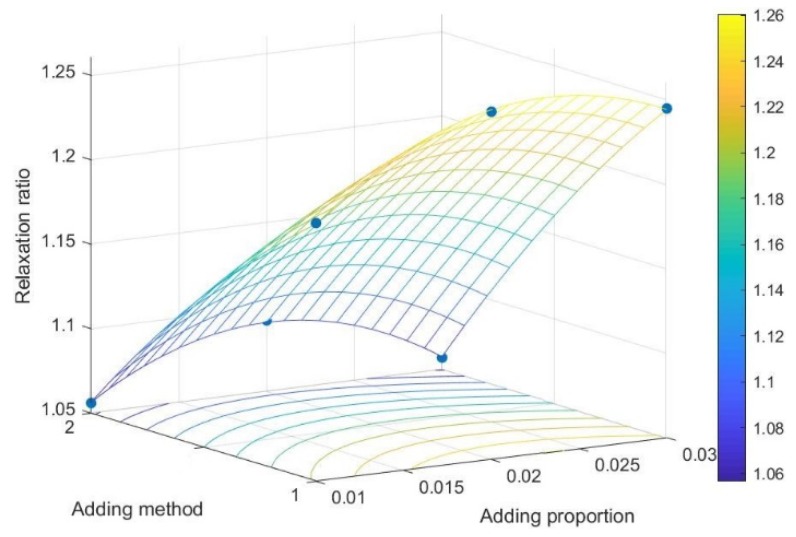
Relation diagram of relaxation ratio–adding proportion and adding method.

It can be seen from Figure 6 that the normalization significance of binder adding method and the proportion of brown sugar water to raw material are 100% and 16.1%, respectively. The two major factors are selected, and the relationship between the relaxation ratio and the two major factors is plotted as shown in Figure 7.

The aim of binder addition using a spraying method is to add smaller particles more evenly to biomass particles so that the binder particles acting among the biomass particles as solid bridge distribute more evenly and provide more strength to densify the biomass particles. Therefore, the value of relaxation ratio of pellets obtained by spraying method is smaller than that obtained by stirring method.

The relaxation ratio is the ratio of the maximum compaction density to the relaxation density of the pellets after densification, and it has certain significance for studying the quality characteristics of the pellets [35]. The smaller the relaxation of the pellets is, the greater the relaxation density will be. Generally, it indicates the better stability, the higher compactness, as well as greater physical strength and retention degree [36]. From the qualitative analysis in Figure 7, it can be known that within the range of factors specified in the test, the proportion of brown sugar water to raw material and brown sugar water by spraying is lower, and the relaxation ratio is smaller. 

The relaxation ratio K_1_ of biomass pellets obtained by stirring method is 1.23467, while the relaxation ratio K_2_ of biomass pellets obtained by spraying method is 1.06833, and range value R = K_1_ − K_2_ = 0.16634. Which means, the relaxation ratio of biomass pellets obtained by spraying method is reduced by R/K_1_ × 100% = 13.47% compared with that by stirring method. 

**Figure 8:**
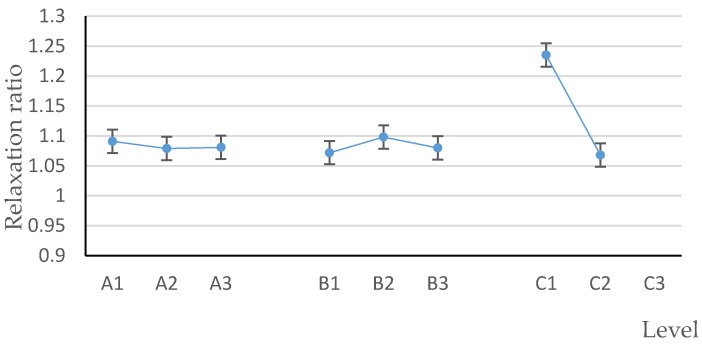
Factors and levels–relaxation ratio.

According to the mean value of each level of each factor (Figure 8), A_2_ is the optimal level of factor A, B_1_ is the optimal level of factor B, and C_2_ is the optimal level of factor C. Therefore, the optimal combination of factors affecting the relaxation ratio is A_2_B_1_C_2_; that is, the optimal conditions for obtaining the optimal relaxation ratio are as follows: The mass ratio of brown sugar to water is 1.5:1, the proportion of brown sugar water to raw material is 1%, and the binder adding method is spraying.

### 3.3. Durability

**Figure 9:**
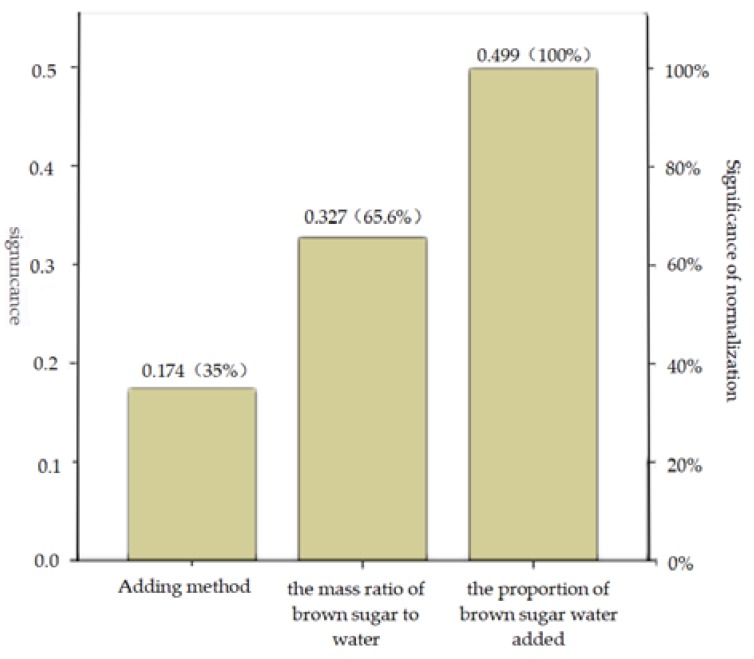
Chart of factor significance.

Table 3 shows the average and range of durability of each factor at different levels. According to the range analysis, the influence degree of each factor on the durability was as follows: the proportion of brown sugar water added > the mass ratio of brown sugar to water > binder adding method. The importance of each factor according to the BP neural network is consistent with the normalized significance (Figure 9) and the range analysis results.

**Figure 10:**
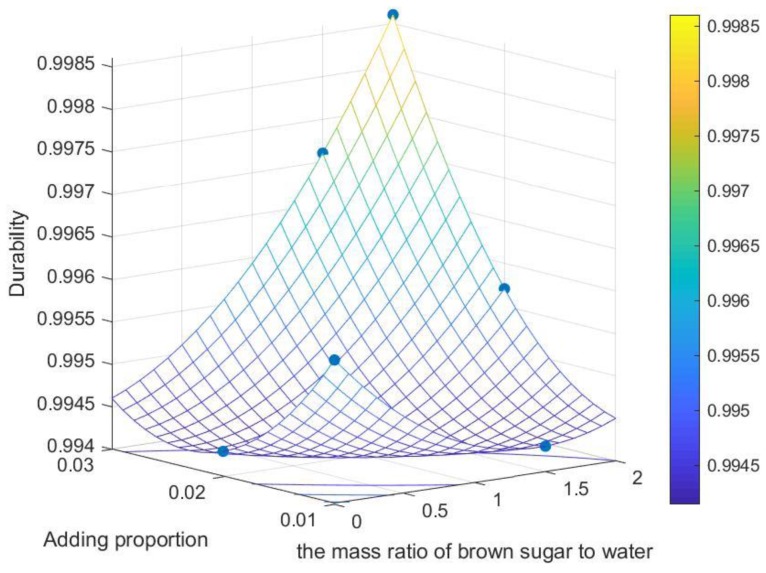
Relation diagram of durability–add proportion and the mass ratio of brown sugar to water.

It can be seen from Figure 9 that the normalized significance of the proportion of brown sugar water added and the mass ratio of brown sugar to water are 100% and 65.6%, respectively. The two major factors are selected, and the relationship between durability and the two major factors is plotted as shown in Figure 10.

**Figure 11:**
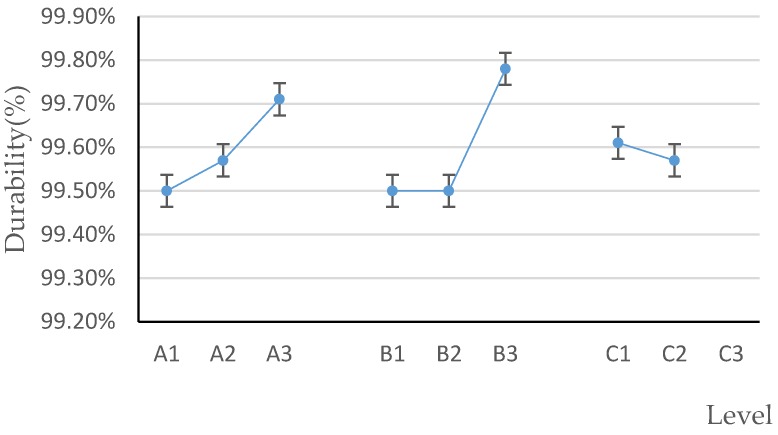
Factors and levels–durability.

Durability is closely related to transportation requirements of biomass pellets [37]. From the qualitative analysis in Figure 10, it can be known that within the range of factors specified in the test, the higher the brown sugar water addition ratio and the higher the mass ratio of brown sugar to water is, the greater the durability will be. According to the mean value of each level of each factor (Figure 11), A_3_ was the optimal level of factor A, B_3_ was the optimal level of factor B, and C_1_ was the optimal level of factor C. Therefore, the optimal factors affecting the level of pellet durability was A_3_B_3_C_1_; that is, the optimal pellet durability is obtained as follows: the mass ratio of brown sugar to water is 2:1, the proportion of brown sugar water to raw material is 3%, and the binder adding method is stirring.

Considering the best combination of three physical indexes, the optimal densification conditions of biomass based on brown sugar water as binder using the spraying method were obtained by comprehensive equilibrium method, which is adopted to analyze and determine the frequency of occurrence of each factor at each level in the optimal combination of the three physical indicators: A_3_B_3_C_2_; that is, mass ratio of brown sugar to water 2:1, proportion of brown sugar water to raw material 3%, and using binder spraying method. The spraying method can mix the binder with the biomass material more evenly when brown sugar water binder was spraying into pine sawdust in the form of tiny particles. Therefore, relaxation ratio of pellets made by spraying method was reduced by 13.47%, and the relaxation density and the quality of biomass pellets were significantly improved.

## 4. Conclusions

This study conducted a comparative biomass densification by two binder adding methods using pine sawdust as raw material and brown sugar water as binder. Through orthogonal experiments, the physical properties (compressive strength, relaxation ratio, durability) of biomass pellets were analyzed, and the optimal densification conditions of biomass pellets were investigated. The optimal densification conditions of pine sawdust were when the compaction pressure was 100 MPa, the mass ratio of brown sugar to water was 2:1, the proportion of brown sugar water to biomass material was 3%, and the binder adding method was spraying. Through the range analysis, it is concluded that the brown sugar water binder can be mixed with the biomass raw material more evenly by the spraying method; thus, the relaxation ratio of pellets derived from spraying method is reduced by 13.47%. The results showed that spraying binder method would bring significant improvement on the relaxation density of the biomass pellets. The study of this paper can support researchers seeking effective ways of adding binders to biomass raw material to improve the quality of the pellets.

## Figures and Tables

**Table 1:** Orthogonal test table of three factors mixed level.

Level	Factor		
	The Mass Ratio of Brown Sugar to Water	The Proportion of Brown Sugar Water Added (%)	Binder Adding Method
1	0:1	1	Stirring
2	1.5:1	2	Spraying
3	2:1	3	

**Table 2:** Test results.

Test Group	Factor and Levels			Test Index		
	A	B	C	Y_1_ (MPa)	Y_2_	Y_3_ (%)
1	1	1	1	0.516 ± 0.56	1.203 ± 0.04	99.57 ± 0.45
2	1	2	2	0.515 ± 0.43	1.092 ± 0.01	99.43 ± 0.38
3	2	3	1	0.469 ± 0.47	1.245 ± 0.02	99.71 ± 0.42
4	2	1	2	0.655 ± 0.65	1.056 ± 0.01	99.43 ± 0.51
5	3	2	1	0.625 ± 0.58	1.256 ± 0.04	99.57 ± 0.49
6	3	3	2	0.758 ± 0.55	1.057 ± 0.03	99.86 ± 0.39

**Table 3:** Range analysis table.

Index		Factors		
		A	B	C
**Y_1_**	K_1_	0.515 ± 0.50	0.585 ± 0.61	0.537 ± 0.54
	K_2_	0.562 ± 0.56	0.570 ± 0.51	0.643 ± 0.54
	K_3_	0.692 ± 0.57	0.614 ± 0.51	
	R	0.176 ± 0.53	0.044 ± 0.56	0.106 ± 0.56
Y_2_	K_1_	1.0905 ± 0.03	1.0725 ± 0.03	1.23467 ± 0.05
	K_2_	1.0795 ± 0.02	1.0985 ± 0.03	1.06833 ± 0.03
	K_3_	1.0810 ± 0.04	1.0800 ± 0.03	
	R	0.01100 ± 0.02	0.02600 ± 0.03	0.16634 ± 0.04
Y_3_	K_1_	0.99500 ± 0.42	0.99500 ± 0.48	0.99617 ± 0.45
	K_2_	0.99570 ± 0.47	0.99500 ± 0.44	0.99573 ± 0.43
	K_3_	0.99715 ± 0.44	0.99785 ± 0.405	
	R	0.00215 ± 0.43	0.00285 ± 0.42	0.00043 ± 0.44

## Appendix A

**Table A1:** Raw data of the pellets.

**Test Group**	**Number**	**Diameter (mm)**	**Length (mm)**	**Mass (g)**	**Maximum Compaction Density (g/cm^−3^)**	**Standard Deviation**
1	pellet1	16.45	32.51	7.02	1.016	0.029512906
	pellet2	16.46	34.89	7.01	0.944	
	pellet3	16.45	33.31	6.99	0.987	
2	pellet1	16.43	35.81	7.03	0.926	0.003575869
	pellet2	16.44	35.85	6.98	0.917	
	pellet3	16.44	35.86	7.02	0.922	
3	pellet1	16.39	34.55	7.02	0.963	0.006132787
	pellet2	16.38	34.29	7.01	0.97	
	pellet3	16.38	34.68	6.98	0.955	
4	pellet1	16.37	35.01	7.03	0.954	0.004848876
	pellet2	16.38	34.98	7.01	0.951	
	pellet3	16.42	34.97	6.98	0.943	
5	pellet1	16.43	35.05	7.03	0.946	0.003325193
	pellet2	16.42	34.78	6.97	0.946	
	pellet3	16.45	34.97	6.98	0.939	
6	pellet1	16.5	35.43	6.98	0.93	0.001485221
	pellet2	16.49	35.45	7.01	0.933	
	pellet3	16.47	35.41	7.02	0.934	
**Test Group**	**Number**	**Diameter (mm)**	**Length (mm)**	**Mass (g)**	**Relaxation Density (g/cm^−3^)**	**Standard Deviation**
1	pellet1	16.46	40.6	7.02	0.813	0.001290021
	pellet2	16.46	40.59	7.01	0.812	
	pellet3	16.45	40.63	6.99	0.809	
2	pellet1	16.44	39.25	7.03	0.844	0.002455449
	pellet2	16.44	39.25	6.98	0.838	
	pellet3	16.45	39.27	7.02	0.841	
3	pellet1	16.54	41.8	7.02	0.782	0.008668709
	pellet2	16.73	41.92	7.01	0.761	
	pellet3	16.66	41.69	6.98	0.768	
4	pellet1	16.5	36.29	7.01	0.903	0.00636098
	pellet2	16.47	36.34	7.03	0.908	
	pellet3	16.56	36.35	6.99	0.893	
5	pellet1	16.52	42.98	7.03	0.763	0.002467339
	pellet2	16.52	42.89	6.97	0.758	
	pellet3	16.54	42.88	6.98	0.758	
6	pellet1	16.5	37.29	6.98	0.875	0.004147738
	pellet2	16.49	37.25	7.01	0.881	
	pellet3	16.47	37.21	7.02	0.886	
